# Macrophage Activation Syndrome in Kawasaki Disease: Insights from a Systematic Literature Review on Diagnosis, Clinical Features, and Treatment

**DOI:** 10.3390/children12030349

**Published:** 2025-03-11

**Authors:** Giulia Inguscio, Stefano Romano, Maria Vincenza Mastrolia, Gabriele Simonini, Teresa Giani

**Affiliations:** 1 Pediatric Department, School of Sciences of Human Health, University of Florence, 50121 Florence, Italy; 2 Rheumatology Unit, ERN ReCONNET Center, AOU Meyer Children’s Hospital IRCCS, 50139 Florence, Italygabriele.simonini@unifi.it (G.S.)

**Keywords:** macrophage activation syndrome, Kawasaki disease, IVIG resistance, refractory Kawasaki disease, splenomegaly, hyperferritinemia

## Abstract

Background: Macrophage activation syndrome (MAS) is a hyperinflammatory and potentially fatal complication associated with rheumatologic disorders. In Kawasaki disease (KD), MAS is a rare and poorly described condition, making its differentiation from a severe, treatment-resistant presentation of KD particularly challenging. Objective: We aimed to describe MAS in KD by analyzing its epidemiological, clinical, and laboratory characteristics, complications, therapeutic strategies, and outcomes. Methods: A comprehensive literature review of PubMed, Embase, Scopus, and Cochrane Library was conducted to identify English-language studies on KD complicated by MAS, including case reports and case series, until 15 November 2024. Results: A total of 176 pediatric patients (60 females; median age 4 years, range 0.13–17) from 48 articles were included. MAS occurred after or simultaneously with KD diagnosis in 174/176 cases (99%). Common features included fever (100%), splenomegaly (49.4%), and hyperferritinemia (98.2%). Cardiac involvement was reported in 37% of children. The HLH-2004 criteria were met in 63% of cases, while the 2016 Ravelli criteria for MAS complicating systemic juvenile idiopathic arthritis were met in 94%. Treatment included additional doses of IVIG (36.2%), GCs (82.8%), cyclosporine A (28.7%), and biologics (13.8%), with complete MAS resolution in 93% of cases. Conclusions: MAS in KD is a rare but severe complication, with overlapping features that make its differentiation from severe and resistant KD challenging. Persistent fever despite initial IVIG administration, along with splenomegaly and hyperferritinemia, emerge as key warning signs. Ravelli criteria provide stronger diagnostic support compared to the HLH-2004 criteria. Moreover, MAS is associated with increased cardiac involvement.

## 1. Introduction

Kawasaki disease (KD) is an acute systemic vasculitis of the medium-sized vessels, primarily affecting children younger than 5 years old [1]. It is characterized by fever, polymorphous skin rash, non-exudative conjunctivitis, erythema of the lips and oral cavity, changes in the extremities, and lymphadenopathy [1]. The most common complication is cardiac involvement, particularly coronary artery lesions (CALs), which occur in approximately 15–25% of untreated patients [1]. Children refractory to first-line treatment with intravenous immunoglobulin (IVIG) are at an increased risk of developing CALs [2].

Macrophage activation syndrome (MAS) is a severe and potentially fatal complication, also referred to as hemophagocytic lymphohistiocytosis (HLH), secondary to rheumatic diseases. It is a hyperinflammatory condition held by the activation and expansion of T cells and macrophages [3], leading to the overproduction of cytokines, especially interferon (IFN)-γ, interleukin (IL)-18, and tumor necrosis factor (TNF)-α [4]. The exact pathogenetic mechanisms of MAS remain unclear, and knowledge regarding its pathogenesis is primarily derived from the genetic forms of HLH. Primary HLH is caused by defects in various genes in which the products are involved in the cytolytic pathway, leading to an uncontrolled expansion of T cells and macrophages [3]. In particular, impaired cytotoxic CD8+ T cell function and disrupted communication between antigen-presenting cells (APCs) and CD8+ T cells fail to properly regulate the immune response, leading to excessive T cell activation. Consequently, macrophages and T lymphocytes produce uncontrolled levels of proinflammatory cytokines, contributing to organ damage and hematologic abnormalities [5]. Notably, heterozygous mutations in primary HLH-related genes (PRF1, LYST, RAB27A, UNC13D, STXBP2, or STX11) have been identified in up to 40% of patients with MAS [6]. In many cases, MAS likely arises from a combination of genetic predisposition, an underlying inflammatory condition, and external triggers like infections or medications [5].

MAS is clinically marked by unremitting high fever, hepatosplenomegaly, lymphadenopathy, cytopenia of all lines of blood cells, impaired liver function, intravascular coagulation, and neurologic dysfunction [7]. MAS is the typical complication of systemic juvenile idiopathic arthritis (s-JIA), affecting approximately 10% of patients with this condition [8]. It can also be associated with other rheumatic diseases, such as Systemic Lupus Erythematosus [9,10]. MAS in KD is rare, although it may be underestimated, as some patients failing to respond to a second dose of IVIG could present a subclinical or “occult” form of the condition [11]. Early recognition and treatment are mandatory, but the overlap in clinical features—such as fever, rash, and liver dysfunction—makes it challenging to distinguish MAS from a severe/refractory KD [12].

The HLH-2004 criteria, developed by the Histiocyte Society [13] for primary HLH, requires at least five out of eight specified criteria: (a) fever; (b) splenomegaly; (c) cytopenia in at least two cell lines; (d) hypertriglyceridemia (>265 mg/dL or 3.0 mmol/L) and/or hypofibrinogenemia (≤150 mg/dL); (e) ferritin > 500 ng/mL; (f) soluble IL-2 receptor (sCD25) > 2400 U/mL; (g) decreased or absent natural killer (NK) cell activity; or (h) hemophagocytosis detected in bone marrow, liver, or lymph nodes. Initially adopted in rheumatology, these criteria have shown a lack of sensitivity for diagnosing MAS in s-JIA [14]. To address this limitation, Ravelli et al. [15] introduced the 2016 consensus criteria specific for MAS in s-JIA, which have also been suggested as potentially applicable for diagnosing MAS in patients with KD [16]. According to these guidelines, a positive diagnosis of MAS is if a patient shows ferritin > 684 ng/mL and satisfies any two of the following: (a) platelet count ≤ 181 × 10^9^/L; (b) aspartate aminotransferase (AST) > 48 U/L; (c) triglycerides > 156 mg/dL; or (d) fibrinogen ≤ 360 mg/dL.

An important differential diagnosis with severe KD is represented by the multisystem inflammatory syndrome in children (MIS-C); MIS-C was identified during the COVID-19 pandemic as a new immune-mediated hyperinflammatory condition linked to SARS-CoV-2 infection or exposure. It shares overlapping features with KD, Kawasaki shock syndrome (KSS), and MAS, such as fever, skin rash, conjunctivitis, and oral mucosal changes [17]. Cattalini et al. [18] characterized clinical symptoms and treatment outcomes of MIS-C in comparison to KD. MIS-C patients showed older age at disease onset, presented more frequent gastrointestinal and respiratory symptoms, higher markers of inflammation, lower white cells, and platelets. Moreover, cardiac involvement is different in the two entities: in patients with KD, the primary complication is the development of CALs, whereas MIS-C patients are more susceptible to myocarditis, leading to heart insufficiency. On the other hand, key characteristics of MAS—such as hypofibrinogenemia, splenomegaly, hypertriglyceridemia, and bone marrow hemophagocytosis—are not typically associated with MIS-C.

MAS in the context of KD is rare and has no standardized treatment. Management is based on experience from other diseases and personal expertise, including treatments such as GCs, IVIG, cyclosporine, and/or biologic drugs [3].

The study aims to comprehensively describe MAS in KD by reviewing current literature. It evaluates epidemiological patterns, clinical features, laboratory findings, complications, treatments, and patient outcomes to improve understanding, promote early recognition, and report current management strategies for this rare but severe complication.

## 2. Material and Methods

We conducted a comprehensive systematic literature review using PubMed, Scopus, Embase, and the Cochrane Library, according to the Preferred Reporting Items for Systematic Reviews and Meta-Analyses guidelines (PRISMA), to identify cases of KD complicated by MAS, including English-language studies published up to 15th November 2024. The keywords used for the research were (“Kawasaki disease” OR “Kawasaki syndrome” OR “Mucocutaneous Lymph Node Syndrome”) AND (“macrophage activation syndrome”, OR “hyperinflammation”, OR “systemic inflammatory response”, OR “Hemophagocytic syndrome” OR “hemophagocytic lymphohistiocytosis”, OR “HLH” OR “MAS”).

The inclusion and exclusion criteria were defined following the PICO framework (Population, Intervention, Comparison, Outcome) to ensure a systematic and transparent selection process. The Population (P) includes children under 18 years old with a diagnosis of KD (based on AHA criteria [1]) and MAS (based on HLH-2004 criteria [13], 2016 Ravelli criteria [15], or clinical opinion). Intervention (I) is aimed at documenting clinical and laboratory variables as well as therapeutic strategies described in the literature. Comparison (C) is not applicable, as the study focuses on descriptive and observational data. Outcome (O) includes complications, resolution, and mortality.

Data were extracted using a pre-defined and standardized form, capturing the following variables: demographic data, clinical characteristics (including presentation, cardiac involvement, and laboratory findings), type and sequence of therapies used, and patient outcomes. Eligible studies included case reports, case series, and observational studies. Articles with aggregated data that did not allow specific extraction for KD and MAS or those lacking diagnostic or therapeutic details to support the analysis were excluded.

Two independent reviewers (S.R. and G.I.) screened titles and abstracts for relevance. Full texts of potentially eligible studies were assessed for inclusion. Discrepancies were resolved through discussion or consultation with a third reviewer (T.G.). The selection process was documented using a PRISMA flow diagram.

## 3. Results

We found 1600 articles; 394 duplicate studies were removed following the initial search. Of these, 48 articles (33 case reports and 15 case series) met the inclusion criteria and were included in this review [4,9,10,12,17,19,20,21,22,23,24,25,26,27,28,29,30,31,32,33,34,35,36,37,38,39,40,41,42,43,44,45,46,47,48,49,50,51,52,53,54,55,56,57,58,59,60,61].

**Figure 1:**
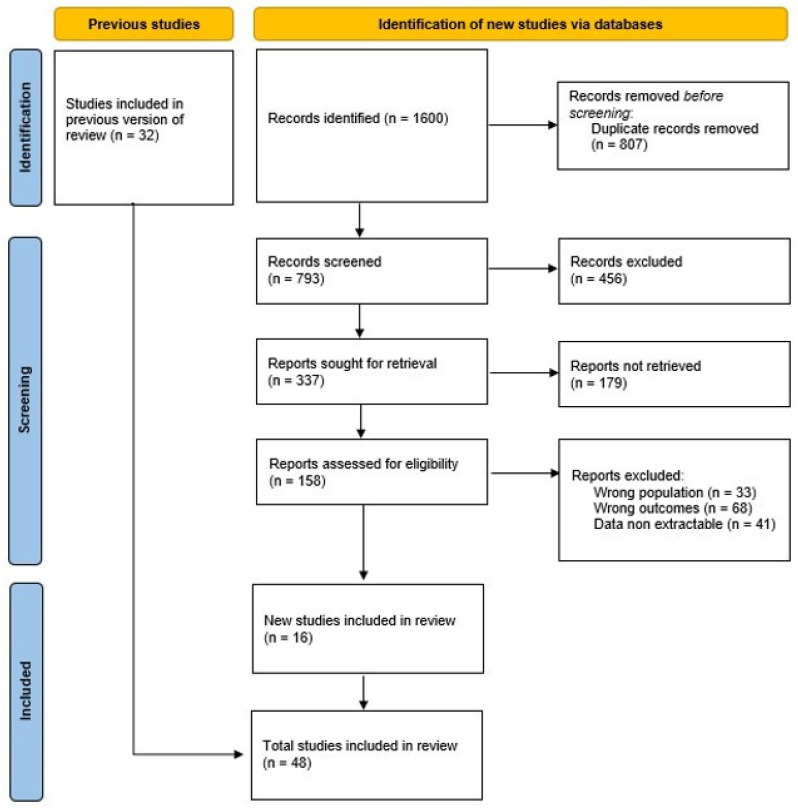
Study flow diagram summarizing the results of the literature search.

The selection process is summarized in the PRISMA flow diagram (Figure 1), detailing the number of articles identified, screened, excluded, and included in the final analysis.

A total of 176 from 48 articles were included in this study; their demographic and clinical characteristics are listed in Table 1. Sixty out of one hundred seventy-six were females (34.1%), with a mean and median age of 5.2 and 4 years, respectively (range 0.13–17 years). One hundred seventy-four out of one hundred seventy-six patients (98.9%) developed MAS either after or simultaneously with the diagnosis of KD. The mean time from symptom onset to MAS diagnosis was 13.4 days (range 5–35 days), and the mean time from KD diagnosis to MAS diagnosis was 7.05 days (range 0–24 days).

Eighty-seven out of one hundred thirty-nine cases (62.6%) had a classic KD presentation, fifty out of one hundred thirty-nine (36%) had an incomplete presentation, and two out of one hundred thirty-nine (1.4%) had an atypical presentation. In thirty-seven cases, the presentation was not reported. Thirteen out of one hundred seventy-six patients (7.4%) developed a KSS.

Cardiac involvement was described in 59/159 cases (37.1%), of whom 22 were classified as having (CAA) aneurysms, 15 coronary ectasia, 1 pericarditis, 2 pericardial effusion, and 1 myocardial infarction. Of this, 3 patients out of 59 with CAA are reported to have persistent lesions at the follow-up [21,24,37]. In 30 patients, cardiac involvement was not specified.

For the diagnosis of MAS, in 81/146 cases (55.5%) the authors used the HLH-2004 criteria, in 57/146 (39%) the 2016 Ravelli criteria were used, and in 8/146 (5.5%), both criteria were used. The HLH-2004 criteria were satisfied in 75/119 (63%, not applicable in 57 patients), while the 2016 Ravelli criteria were satisfied in 160/170 cases (94.1%, not applicable in 6 patients).

The most frequent clinical characteristic was fever (176/176, 100%), followed by splenomegaly (87/176, 49.4%) and hepatomegaly (51/115, 44.3%). Other clinical characteristics were central nervous system (CNS) involvement (27/115, 23.5%), respiratory distress (22/115, 19.1%), bleeding complications (15/115, 13%), renal involvement (11/115, 9.6%) and BCG (*Bacillus Calmette-Guerin*) scar redness (3/115, 2.6%).

Hyperferritinemia was present in 98.2% of cases (median value 2376 ng/mL), bi-trilinear cytopenia in 57.1%, and hypertriglyceridemia (>265 mg/dL) and/or hypofibrinogenemia (≤150 mg/dL) in 55.3% (median value 247.5 mg/dL and 175 mg/dL, respectively).

Hemophagocytosis was detected in 70/123 patients (56.9%), low or absent NK cell activity in 18/36 (50%), and increased levels of sCD25 in 7/10 (70%); in the remaining patients, data were not available. The mean, median, and range values of laboratory data are listed in Table 2.

KD first-line treatment with IVIG was reported in 174/176 patients (98.8%), and 63/174 (36.2%) received a second dose of IVIG. Adjunctive therapies for fever persistence were GCs in 144/174 cases (82.8%), cyclosporine A in 50/174 (28.7%), chemotherapy in 36/174 (20.7%, of which 35 were treated with etoposide, and 1 was treated with cyclophosphamide), and biologic therapies in 24/174 (13.8%) (anakinra in 6 cases, and infliximab in 18), and methotrexate in 1/174 (0.6%). In 139/150 patients (92.7%), a complete resolution of the inflammatory status was reported. Eleven patients (6.25%) died; for the remaining 26 children, no data were available. The treatment data are summarized in Table 3.

## 4. Discussion

MAS is a potentially fatal complication that can arise during various rheumatic diseases, characterized by an uncontrolled immune response and excessive production of proinflammatory cytokines [62]. MAS in KD is a rare event reported in 0.9–1.9% [9,10] across different populations. Zhang et al. [25] and Rhee et al. [20] reported a prevalence of 0.9% in their Eastern Asian cohorts, consisting of 3186 patients with KD (28 with MAS) and 468 patients with KD (4 with MAS), respectively. In contrast, Latino et al. [19] observed a higher prevalence of 1.9% (12 out of 638 patients) in a Canadian population.

We analyzed 176 patients across 48 articles on pediatric KD complicated by MAS. The age of the patients in this cohort ranged from infants (1 month old) to teenagers (17 years old), with a median age of 4 years, which is older compared to the typical age at onset of KD. A Japanese survey (2015–2016) of 31,595 patients found the highest KD incidence in children aged 9–11 months [63], while European studies report a mean onset age between 1.9 and 2.8 years [64,65,66,67,68]. This aligns with findings from the 2017 review by García-Pavón et al. [9], which reported a median age of 5.6 years. Some authors [69] have suggested older age to be a risk factor for MAS. Additionally, we observed an unequal sex distribution, with nearly two-thirds of cases (66%) being male, in accordance with García-Pavón et al. [9] reporting a male prevalence of 68%. Experimental research on murine models demonstrated that males exhibit a stronger IL-1 mediated inflammatory response compared to females [70] suggesting that males are not only more affected by KD but also experience a more severe disease course.

Most patients with KD complicated by MAS presented with classic KD features (62.6%), characterized by fever and at least four typical signs. In nearly all cases, MAS developed concurrently with or shortly after KD onset. Two exceptions were noted: in one case, KD was diagnosed two days after MAS onset [59], and in the other, the diagnosis was made post-mortem based on histopathological findings of CALs [58].

While CALs are observed in 20% to 25% of patients with untreated KD, decreasing to 4% to 6% with IVIG treatment [71,72], this percentage increases to 37% in patients with KD with MAS, highlighting a more severe disease trajectory.

The hyperinflammatory state associated with MAS, characterized by excessive immune cell activation and cytokine overproduction, likely intensifies vascular damage, thereby elevating the risk of CALs.

Fever was present in 100% of cases, with its persistence despite first-line KD treatment being a typical sign of MAS. Some authors noted that fever lasting more than 10 days is strongly associated with MAS in these patients [29].

Splenomegaly, reported in 49.4% of cases, is the second most frequent clinical feature. Being uncommon in KD, with a reported prevalence of only 1.4% in a cohort of 365 patients with KD [73], its presence serves as a clinical red flag, potentially indicating the onset of MAS, and being associated with a higher incidence of CALs [74]. In contrast, splenomegaly is more common in s-JIA-related MAS, where it was initially included in the HLH-2004 criteria [13] but subsequently excluded in the 2016 consensus criteria [15].

We found nearly a quarter (23.5%) of the patients with KD complicated with MAS to exhibit signs of CNS involvement, such as seizures, irritability, and altered mental status. In the absence of MAS, CNS involvement in KD has been reported to present with a highly variable range of symptoms and incidence that ranges from 1% to 30%, depending on the study [75,76]. A monocentric Chinese study involving 1582 patients reported neurological involvement in 5.1% of KD cases. These patients demonstrated a higher inflammatory burden and an increased resistance to IVIG treatment, suggesting that CNS involvement may represent a sign of disease severity [77].

Historically, the diagnosis of primary HLH was based on the HLH-2004 criteria [13], which were initially designed for primary HLH. While these criteria have been adapted for secondary HLH/MAS, they exhibit several limitations and do not fully align with the specific characteristics of MAS. In 2016, Ravelli consensus criteria were proposed for the diagnosis of MAS in s-JIA and have proven to be well-designed and highly effective in identifying MAS in this context. Subsequently, they have also been adopted in clinical practice within the KD field [16]. We found that the HLH-2004 criteria were applied in 81 out of 146 cases (55.5%), Ravelli’s guidelines in 57 out of 146 cases (39%), and both criteria in 8 out of 146 cases (5.5%). However, the Ravelli criteria have been increasingly utilized in recent years since their publication, likely reflecting a perception among authors that these criteria are more sensitive. Supporting this, the HLH-2004 criteria were fulfilled in 63% of cases, compared to 94% for the 2016 Ravelli criteria. The analysis of sCD25 levels and NK cell activity as indicated in HLH-2004 criteria is not readily available in many hospitals, and evidence of hemophagocytosis is not routinely performed in patients with KD. Moreover, hemophagocytosis alone is neither a definitive nor sufficient criterion for diagnosing MAS, as its presence may reflect the severity of the inflammatory process rather than the underlying cause [3]. In the studies included in our review, bone marrow analysis was performed in 70% of cases, and it was found to be positive in 57% of children, confirming its limited sensitivity as a diagnostic marker for MAS. The 2016 Ravelli criteria seem to offer a more practical and sensitive tool for diagnosing MAS in KD compared to HLH-2004. Their higher detection rate and reliance on more accessible laboratory parameters suggest they may be a preferable choice in clinical settings, even if further prospective studies are needed to validate these findings and optimize MAS diagnosis in patients with KD.

Hyperferritinemia is included in both HLH-2004 and Ravelli criteria [3]. While in the HLH-2004 criteria, hyperferritinemia is included as one of the eight diagnostic criteria (ferritinemia ≥ 500 ng/mL), its critical role is emphasized in the 2016 Ravelli guidelines, where it serves as an entry criterion, necessary for the diagnosis, with a higher cut-off level (≥684 ng/mL). Based on this cut-off, 97% of patients were found to have hyperferritinemia, suggesting a key role for this laboratory finding even within the context of KD.

Changes in other laboratory parameters, especially platelet count, transaminases, and lactate dehydrogenase (LDH), triglycerides and fibrinogen are highly sensitive t identifying MAS in patients with s-JIA [78]. Blood tests for these parameters are widely accessible, making them suitable in clinical practice also in KD [3].

The cut-off levels for triglycerides and fibrinogen are different in the two guidelines, respectively, >265 mg/dL and ≤150 mg/dL for the HLH-2004 criteria and >156 mg/dL and ≤360 mg/dL for the 2016 consensus criteria. Hypertriglyceridemia and hypofibrinogenemia were identified in 48/160 (30%) and 36/148 (24.3%) of patients, respectively, based on HLH-2004 criteria, and in 116/160 (72.5%) and 128/148 (86.5%) of patients, respectively, according to the 2016 Ravelli guidelines.

From the reviewed cases, we observed high LDH levels (median value 1190 U/L), and although nonspecific for MAS, they reflect cellular damage and metabolic reprogramming triggered by stress and inflammatory conditions. In 2021 Roh et al. [30] 28 also reported significantly higher LDH levels in patients with KD with MAS compared to those without, with medians of 384.0 U/L (IQR: 256.0–561.5) in uncomplicated KD and 1319.9 U/L (IQR: 748.0–1485.0) in KD with MAS (*p* = 0.001), supporting LDH as a sign of disease severity.

The treatment of MAS complicating KD is particularly challenging, due to the rarity of this condition and the lack of established guidelines. Management strategies are often borrowed from MAS in other diseases. In our literature review, all but two patients received IVIG as first-line therapy for KD. However, due to persistent fever, most required additional treatments, with only three patients responding to a single dose of IVIG as the sole therapy. Patients with KD who develop MAS typically present with prolonged fever despite first-line treatment, making IVIG resistance a critical red flag [11]. However, due to their clinical overlapping, distinguishing MAS and refractory KD may often be challenging [20].

In the 2022 EULAR/American College of Rheumatology task force described points to consider in the early diagnosis and treatment of MAS/HLH. They recommend prompt empiric immunomodulation in suspected HLH/MAS based on the use of glucocorticoids (GCs), the recombinant IL-1 receptor antagonist (IL-1RA) anakinra and/or IVIG [79]. We found GCs to be the standard treatment (82.8%), mainly using pulse methylprednisolone, though dosage and duration varied across studies. This aligns with Baldo et al.’s findings regarding the treatment of MAS in rheumatologic conditions, emphasizing the variability in therapeutic approaches and the need for standardized guidelines [80].

A total of 35 out of 174 patients (20%) were treated with the HLH-2004 protocol [13], which includes etoposide, dexamethasone, and cyclosporin A. However, its use in secondary MAS is debated due to concerns about its aggressiveness [81]. Notably, most deaths occurred in patients managed with this protocol, aligning with García-Pavón et al.’s [9] report, which documented a 29% mortality rate (7/24 patients) in their review of cases treated with the HLH-2004 protocol.

Cyclosporine A, a safe, effective, and affordable treatment [82] also included in the AHA 2024 guidelines for refractory KD [1] and frequently used in MAS secondary to rheumatologic diseases [80], was administered in the HLH-2004 protocol for 32 patients; in 18 patients, it was combined with GCs, methotrexate in 1 case, and anti-TNF biologic therapy in 4 patients.

The main biologic drugs used were anakinra and infliximab. Blockage of IL-1 with anakinra has been demonstrated to be successful and safe in MAS, especially when secondary to sJIA [83]. Several studies report the efficacy of anakinra in treating MAS complicating KD, and this drug has also been included in the AHA 2024 guidelines for refractory KD [1].

Although the role of anti-TNF in MAS is less clear, we found it was more frequently used than anakinra.

Infliximab immunomodulating role in KD has been studied by Koizumi et al. [84] They demonstrated that infliximab in patients with KD regulates activated monocytes and Treg cells. Moreover, according to Jinkawa et al. [51], sTNFR-II levels in patients with KD were higher during the MAS phase compared to the acute phase of the disease.

In this cohort of patients, we did not find a mention of other drugs that have been recently used in recent years for the treatment of s-JIA-related MAS, such as anti-IL6 therapies (tocilizumab), anti-IFN-γ monoclonal antibody (emapalumab) and Janus kinase (JAK) inhibitors (ruxolitinib) [80]. In a mixed cohort of patients with MAS, authors described one case of KD successfully treated with ruxolitinib [85]; however, this patient was not included in our review because the data of the single patient were not extractable.

The fatality rate in KD is usually low, with an in-hospital mortality rate of approximately 0.17% in the United States and an exceptionally low rate of 0.015% in Japan, reflecting the benefits of early diagnosis and prompt treatment [63,86,87]. However, the standardized mortality ratio (SMR) is significantly elevated in patients with CAA, particularly in males, with an SMR of 2.55 [87]. In this literature review, 6.25% of the patients died; however, based on the data provided, it is not possible to determine the overall mortality rate or to make a direct comparison between the mortality rate in KD overall and that in KD complicated by MAS. Three children died of myocardial infarction [12,42,58], two of whom were treated with the HLH-2004 protocol [12,58]. The patient described by Titze et al. [58] received Etoposide and Cyclosporine A without any IVIG administration.

Other causes of death were disseminated intravascular coagulation [25,28,32], pneumonia [28,32], and multiple organ failure [25]. Kim et al. found patients with KD-related MAS to have a poor prognosis compared to MAS secondary to other conditions, mainly related to disease reactivation and death [32]. Some authors described a higher prevalence of CAA (10–50%) in patients with KD and MAS [23,26]. Nevertheless, most patients had no residual CAAs at follow-up. In our review, 3/59 patients with CAA are reported to have residual coronary damage at their last follow-up [21,24,37]. Concentrations of NT-proBNP (N-terminal pro-B-type natriuretic peptide), a marker of cardiac stress, also appear to be higher in patients with KD-MAS compared to those with KD alone [88].

This study has several limitations. Firstly, most of the included studies are case reports with small sample sizes and retrospective designs, highlighting the lack of multicentric studies to enhance generalizability. MAS may also be under-reported, as diagnostic laboratory tests were often performed only in clinically suspected cases, potentially overlooking subclinical or occult presentations. The absence of standardized diagnostic and treatment protocols for MAS in KD contributes to significant data heterogeneity, with studies employing varying criteria such as HLH-2004, 2016 Ravelli guidelines, or clinical judgment. The heterogeneity in treatment protocols and the lack of robust data about long-term outcomes make it difficult to provide an accurate comparison between the different treatments. Additionally, missing data, variability in the timing of data collection, and inconsistencies in reporting further compromise the reliability of the findings. The absence of a comparison group of patients with KD without MAS limits the ability to contextualize results and draw robust conclusions about specific features, risk factors, and outcomes.

## 5. Conclusions

MAS in KD is a rare but severe complication, primarily affecting older children and males. Key features include splenomegaly, neurological symptoms, hyperferritinemia, and resistance to first-line IVIG treatment. The 2016 Ravelli criteria show greater sensitivity than the HLH-2004 criteria, but diagnostic challenges remain. Coronary artery involvement is more frequent in patients with MAS, and the 6.25% mortality rate underscores the condition’s severity. While glucocorticoids remain the cornerstone of treatment, biologic agents offer promising adjunctive options. Standardized diagnostic and therapeutic protocols are needed.

## Figures and Tables

**Table 1:** Demographic and clinical characteristics of 176 Kawasaki disease cases complicated by macrophage activation syndrome.

Age	5.2 y (Mean) 4 y (Median) 0.13–17 y (Range)
Sex	Males 116 (65.9%) Females 60 (34.1%)
Type of KD	Complete 87/139 (62.6%) Incomplete 50/139 (36%) Atypical 2/139 (1.4%)
Fever	176/176 (100%)
Splenomegaly	87/176 (49.4%)
Hepatomegaly	51/115 (44.3%)
Cardiac involvement	59/159 (37.1%)
CNS involvement	27/115 (23.5%)
Respiratory distress	22/115 (19.1%)
Bleeding	15/115 (13%)
Renal involvement	11/115 (9.6%)
BCG scar redness	3/115 (2.6%)

Abbreviations: y: years old; KD: Kawasaki disease; CNS: central nervous system; BCG: Bacillus Calmette-Guerin.

**Table 2:** Laboratory data of 176 Kawasaki disease cases during the acute phase of macrophage activation syndrome.

Laboratory Data	N. Patients	Mean	Median	Range
Hemoglobin (g/dL)	136	9.16	9.4	4.5–9.9
Leucocyte (×10^9^/L)	120	11.598	8640	100–38,300
Neutrophil count (×10^9^/L)	63	6318	2236	96–33,900
Platelet count (×10^6^/L)	149	272,542	88,000	2800–1,213,000
ESR (mm/h)	94	41.2	42	2–110
CRP (mg/dL)	111	10.78	10.6	0.4–32.48
AST (U/L)	115	329.2	186	17–1468
ALT (U/L)	110	249.8	143	10–1099
LDH (U/L)	82	1562.7	1190	205–4487
Albumin (g/dL)	75	2.87	2.8	2.13–3.7
Triglycerides (mg/dL)	160	259.7	247.5	39–956
Fibrinogen (mg/dL)	148	194.4	175	28–678
Ferritin (ng/mL)	170	9468.3	2376	283–121,527

Abbreviations: ESR: erythrocyte sedimentation rate; CRP: C-reactive protein; AST: aspartate aminotransferase; ALT: alanine aminotransferase; LDH: lactate dehydrogenase.

**Table 3:** Therapies used in patients with KD complicated with MAS.

Therapy	N. Patients
IVIG	174/176 (98.8%)
Additional doses of IVIG	63/174 (36.2%)
Corticosteroids	144/174 (82.8%)
Cyclosporine A	50/174 (28.7%)
Etoposide	35/174 (20.1%)
Cyclophosphamide	1/174 (0.6%)
Methotrexate	1/174 (0.6%)
Anakinra	6/174 (3.4%)
Infliximab	18/174 (10.3%)

Abbreviations: IVIG: intravenous immunoglobulins.
